# The Practical Medicine Series

**Published:** 1915-02

**Authors:** Theo. Y. Hull


THE PRACTICAL MEDICINE SERIES. Edited by Charles L. Mix, A. M., M. D., and Robert T. Vaughan, Ph. B., M. D. Volume I, 1914 Series. General Medicine. Edited by Frank Billings, M. S., M. D., and J. H. Salisbury, A. M., M. D.

This excellent review of the year's work in general medicine devotes the first 120 pages to a discussion of infectious diseases. The very careful, thorough and accurate surveys of the subjects of tuberculosis and pneumonia are particularly noteworthy. Particular and deserved attention is given to the study of the physiology and diseases of the heart. Diseases of the blood vessels, the blood and blood-making organs are interestingly discussed. Progress in our knowledge of the ductless glands, the diseases of metabolism, the urine and the diseases of the kidneys is carefully noted. On the whole the volue is a painstaking and handy review of the subjects covered. The size of the volume, the clearness of type, the conciseness of statement, and the keen perception of the real advances in medicine are all characteristics that commend it to the busy physician who wishes to keep abreast of the times.

THEO. Y. HULL, B. S., M. D.

